# MiR-132/212 promotes the growth of precartilaginous stem cells (PCSCs) by regulating Ihh/PTHrP signaling pathway

**DOI:** 10.1042/BSR20191654

**Published:** 2020-05-11

**Authors:** Fu-Yong Zhang, Yun-Fang Zhen, Zhi-Xiong Guo, Jin Dai, Lun-Qing Zhu, Xu Cao, Guang-Hao Su, Wen-Yan Zhang, Jian-Feng Fang, Zhen-Hua Zhu, Chun-Hua Yin, Ya Liu, Gao Yu, Tan-Tan Zhao, Ya Zhang, Quan-Wen Yuan, Xiao-Dong Wang

**Affiliations:** 1 Department of Orthopaedics, Children’s Hospital of Soochow University, Suzhou 215000, China; 2 Department of Urology, Children’s Hospital of Soochow University, Suzhou 215000, China

**Keywords:** hedgehog, Ihh/PTHrP, miR-132/212, precartilaginous stem cells

## Abstract

Precartilaginous stem cells (PCSCs) are adult stem cells that can initiate chondrocytes and bone development. In the present study, we explored whether miR-132/212 was involved in the proliferation of PCSCs via Hedgehog signaling pathway. PCSCs were isolated and purified with the fibroblast growth factor receptor-3 (FGFR-3) antibody. Cell viability, DNA synthesis and apoptosis were measured using MTT, BrdU and flow cytometric analysis. The mRNA and protein expression were detected by real-time PCR and Western blot, respectively. The target gene for miR-132/212 was validated by luciferase reporter assay. Results showed that transfection with miR-132/212 mimic significantly increased cell viability and DNA synthesis, and inhibited apoptosis of PCSCs. By contrast, miR-132/212 inhibitor could suppress growth and promote apoptosis of PCSCs. Luciferase reporter assays indicated that transfection of miR-132/212 led to a marked reduction of luciferase activity, but had no effect on PTCH1 3′-UTR mutated fragment, suggesting that Patched1 (PTCH1) is a target of miR-132/212. Furthermore, treatment with miR-132/212 mimics obviously increased the protein expression of Indian hedgehog (Ihh) and parathyroid hormone related protein (PTHrP), which was decreased after treatment with Hedgehog signaling inhibitor, cyclopamine. We also found that inhibition of Ihh/PTHrP signaling by cyclopamine significantly suppressed growth and DNA synthesis, and induced apoptosis in PCSCs. These findings demonstrate that miR-132/212 promotes growth and inhibits apoptosis in PCSCs by regulating PTCH1-mediated Ihh/PTHrP pathway, suggesting that miR-132/212 cluster might serve as a novel target for bone diseases.

## Introduction

Precartilaginous stem cells (PCSCs), a type of adult stem cells, are discovered in the epiphyseal organ in embryonic limbs. PCSCs play critical roles in endochondral ossification, limb growth, cartilage development and repair of damaged articular cartilage [1,2]. MicroRNAs (miRNAs) are a group of small, noncoding RNAs that play critical roles in diverse biological processes, such as cell growth, differentiation, apoptosis and angiogenesis [3]. It is well known that miRNAs lead to post-transcriptional silencing of their target mRNAs through complementary binding to the 3′ untranslated region (UTR) of mRNA targets. Previous studies have suggested that miRNAs are required for skeletal development using mice with conditional knockdown of Dicer in chondrocytes of the growth plate [4,5]. Accumulating evidence indicates that miRNAs play an important role in regulating multiple developmental processes in diverse organisms such as stem cell chondrogenic differentiation for osteogenic differentiation or articular cartilage regeneration [6]. For instance, miRNAs are required for skeletal development in mice lacking of Dicer, an enzyme required for miRNA biogenesis [7,8]. However, only small subsets of miRNAs have been explored during development of organism cartilage [9].

miR-132 and miR-212, or named as the miR-132/212 cluster, are encoded in the same intron of a non-coding gene on chromosome 17 in humans. Researchers have demonstrated that the miR-132/212 cluster play an important role in the vascular smooth muscle dysfunction [10]. Up-regulation of miR-132/212 cluster inhibits retinoblastoma tumor-suppressor gene (Rb1) expression, contributing to the growth of pancreatic cancer cells [11]. In addition, a recent study shows that miR-132/212 cluster promotes arteriogenesis via modulation of the Ras-MAPK signaling pathway after hindlimb ischemia [12]. In the present study, we for the first time explored the role of miR-132/212 cluster in PCSCs and its underlying mechanism.

## Materials and methods

### Animals

All animal experiments were performed according to protocols approved by the Animal Care and Use Committee of Children’s Hospital of Soochow University. Healthy New Zealand white rabbits with the age of 3 weeks were purchased from Shanghai Laboratory Animals Center. Animals were housed under a 12-h light/dark cycle with unlimited access to food and water. Animal experiments were conducted in Soochow University.No anaesthetics were used in the study.

### Cell culture

The PCSCs were acquired from the cartilage tissue of rabbits using the enzymic methods. To be brief, New Zealand white rabbits were killed by cervical dislocation. The tissues located around the perichondrial mesenchyme were cut down and digested in trypsin and collagenase type II (Sigma, St. Louis, MO, U.S.A.). Cells were dispersed and incubated with fibroblast growth factor receptor-3 (FGFR-3) antibody (1:500, Santa Cruz Biotechnology Inc., Santa Cruz, CA, U.S.A.) and then purified by positive immunomagnetic selection using Mini-MACS (Miltenyi Biotech, Bergisch Gladbach, Germany). Subsequently, cells were maintained in DMEM medium supplemented with 10% FBS, 100 U/ml ampicillin, and 100 U/ml streptomycin, and cultured in an incubator maintained at 37°C with 5% CO_2_. Cells were passaged at 1:5 dilution when they reached 30–60% confluency using trypsin. Cells of generation 2–4 were used for further experiments.

### Measurement of cell viability

Cell viability was measured by MTT assay (MD Millipore, Billerica, MA, U.S.A.). Briefly, cells were cultured at 3 × 10^4^ cells/well in a 96-well culture plates for overnight. Then 3-(4,5-dimethylthiazol-2-yl)-2,5-diphenyltetrazolium bromide (MTT, Sigma, St. Louis, MO, U.S.A.) at a concentration of 5 mg/ml was added to each well at 10 μl/100 μl followed by incubation at 37°C for 2 h in a cell culture incubator. The medium was replaced with 100 μl dimethyl sulfoxide (DMSO), and the absorbance for each well was measured at 570 nm using a scanning multi-well spectrometer (Bio-Tek instruments, Inc., Burlington, VT, U.S.A.). All experiments were performed in triplicate and repeated three times.

### MiRNAs transfection

MiRNAs transfections were performed using Lipofectamine 2000 reagents (ThermoFisher Scientific, Waltham, MA, U.S.A.) according to the manufacturer’s instructions. Briefly, 2 × 10^5^ cells/well were plated in six-well plate for overnight. After refreshing medium, cells were transfected with miRNAs mimic or inhibitor followed by downstream experiments.

### RNA isolation and real-time PCR

Total RNA was extracted using a TRIzol Reagent (Invitrogen, Carlsbad, CA) according to the manufacturer’s instruction. For detection of mRNA expressions, cDNA synthesis was performed from 1 µg total RNA using a SuperScript First-Standard Synthesis system for RT-qPCR (Invitrogen Life Technologies). Real-time PCR was performed by an ABI PRISM 7900 Sequence Detection System (Applied Biosystems, Foster City, CA, U.S.A.). The primers were: Indian hedgehog (Ihh): forward: 5′-CAAGAAGCCCGGGATCTACA-3, reverse: 5′-GCTCGGGACTTTGTTGCTTG-3′; parathyroid hormone related protein (PTHrP): forward 5′-ACGCCCCATACAACAAAATC-3′, reverse 5′-GGTCACTGCTTGTCCAGATG-3′; and β-actin: forward 5′-GGCTGTGCTATCCCTGTACG-3′, reverse 5′-AGGTAGTCAGTCAGGTCCCG-3′. The reactions were incubated in a 96-well optical plate at 95°C for 10 min, followed by 40 cycles at 95°C for 15 s and 60°C for 1 min. Relative expressions were calculated by the 2^−△△CT^ method.

### Bioinformatic analyses

The mature sequence of miR-132/212 was from the miRNA database (http://www.mirbase.org/). miRanda (http://www.microrna.org), PicTar (http://pictar.mdc-berlin.de/) and TargetScan (http://www.targetscan.org/) were used to predict the target gene of miR-132/212.

### Luciferase assay

Luciferase reporter assay was done by transient co-transfection of psiCHECK2-report vector (Ambion, Applied Biosystems, Foster City, CA, U.S.A.) containing wild-type or mutant 3′UTR of human PTCH1 containing the putative miR-132/212 cluster binding site into 293T cells in 24-well plates using Lipofectamine 2000 (Invitrogen) according to the manufacturer’s instructions. For luciferase reporter assay, 48 h after transfection, luciferase activity was measured using the Dual-Luciferase Reporter Assay System (Promega, Madison, WI, U.S.A.). Luciferase activity was read by SpectraMax M5 (Molecular Devices, Sunnyvale, CA, U.S.A.). Experiments were performed in triplicate and repeated three times.

### Western blot

Total proteins were extracted from PCSCs, and protein doses were measured using BCA kit (Pierce, Rockford, IL). Protein samples were separated by SDS-PAGE and transferred to nitrocellulose membranes. The membranes were incubated with primary antibodies against Ihh (1:1000, Santa Cruz Biotechnology Inc., Santa Cruz, CA, U.S.A.) and PTHrP (1:1000, Santa Cruz Biotechnology Inc., Santa Cruz, CA, U.S.A.) overnight. Then, the membrane was incubated with appropriate horseradish peroxides–conjugated secondary antibodies at room temperature for 1 h. Protein bands were detected using a Super Signal Enhanced Chemiluminescence kit (Pierce, Rockford, IL, U.S.A.).

### Statistical analysis

Statistical evaluation for data analysis was determined by unpaired Student’s *t* test. All data were shown as the means. A statistical difference of *P*<0.05 was considered significant.

## Results

### Isolation, purification and identification of PCSCs

**Figure 1:**
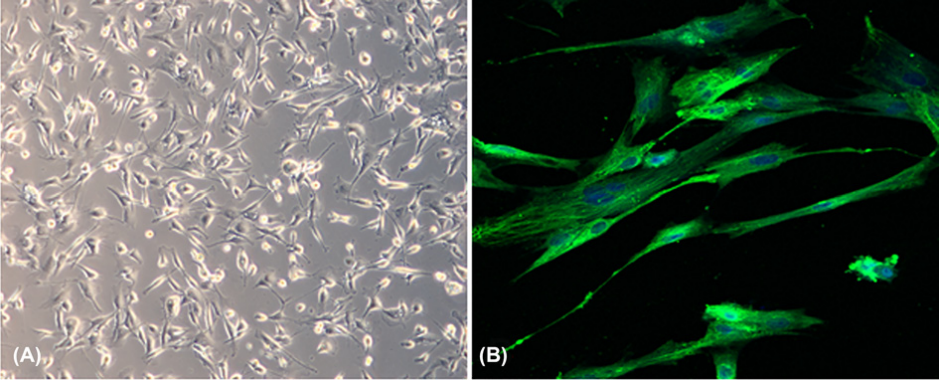
Isolation and identification of PCSCs PCSCs were isolated from the neonate rabbits’ distal epiphyseal growth plate and the morphology of PCSCs were observed under light microscope (**A**) and immunostaining with FGFR-3 (**B**).

PCSCs were successfully isolated from the neonate rabbits’ distal epiphyseal growth plate using the methods described above. The morphological images of PCSCs were shown either under light microscope (Figure 1A) and immunostaining (Figure 1B). Fibroblast growth factor receptor-3 (FGFR-3) was recognized as a marker for PCSCs. Therefore, we detected its expression in the cultured PCSCs. The immunofluorescence image suggested positive FGFR-3 expression in PCSCs.

### miR-132/212 cluster promotes growth and DNA synthesis of PCSCs

**Figure 2:**
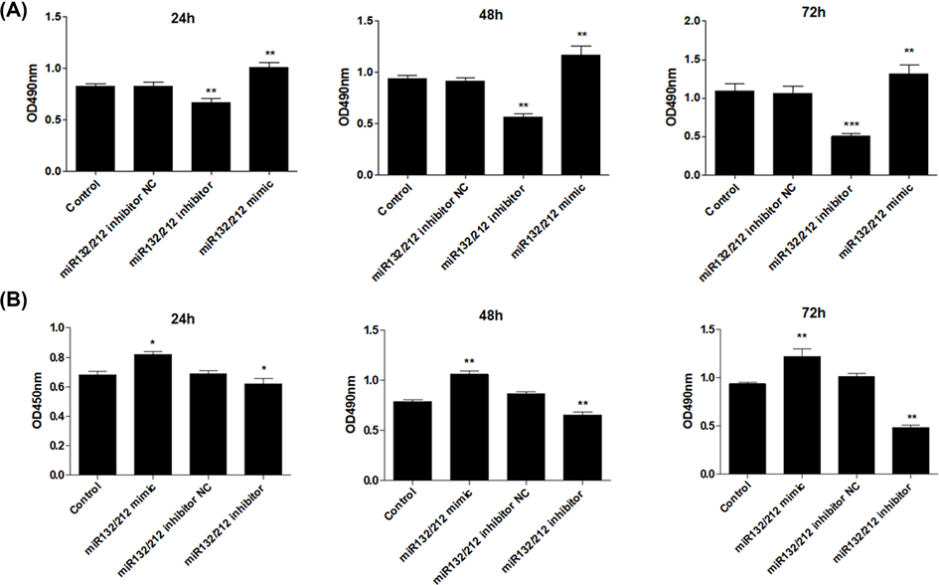
miR-132/212 cluster promotes growth and DNA synthesis of PCSCs After transfection with miR-132/212 mimic, inhibitor and negative control (NC), MTT assay (**A**) and BrdU assay (**B**) were performed to measure the cell viability and DNA synthesis of PCSCs at 24, 48 and 72 h; *P<0.05, **P<0.01.

In order to investigate the role of miR-132/212 cluster in the cell viability of PCSCs, miR-132/212 mimic, inhibitor and negative control (NC) were transfected into PCSCs and cultured for different time points. MTT analysis showed that miR-132/212 mimic transfection for 24 h slightly, but significantly, increased cell viability of PCSCs. By contrast, miR-132/212 inhibitor suppressed PCSCs growth (Figure 2A). miR-132/212 inhibitor NC had no obvious effects on PCSCs growth. At 48 and 72 h, overexpression of miR-132/212 cluster further enhanced cell growth of PCSCs. Conversely, inhibition of miR-132/212 cluster decreased PCSCs growth (Figure 2A).

Next, we explored the role of miR-132/212 cluster in DNA synthesis of PCSCs using BrdU assay. After transfected, we found that up-regulation of miR-132/212 cluster for 24 h promoted the DNA synthesis of PCSCs (Figure 2B). Meanwhile, overexpression of miR-132/212 cluster further enhanced DNA synthesis of PCSCs. However, transfection with miR-132/212 inhibitor suppressed DNA synthesis in PCSCs in a time-dependent manner (Figure 2B).

### miR-132/212 cluster suppresses apoptotic death in PCSCs

**Figure 3:**
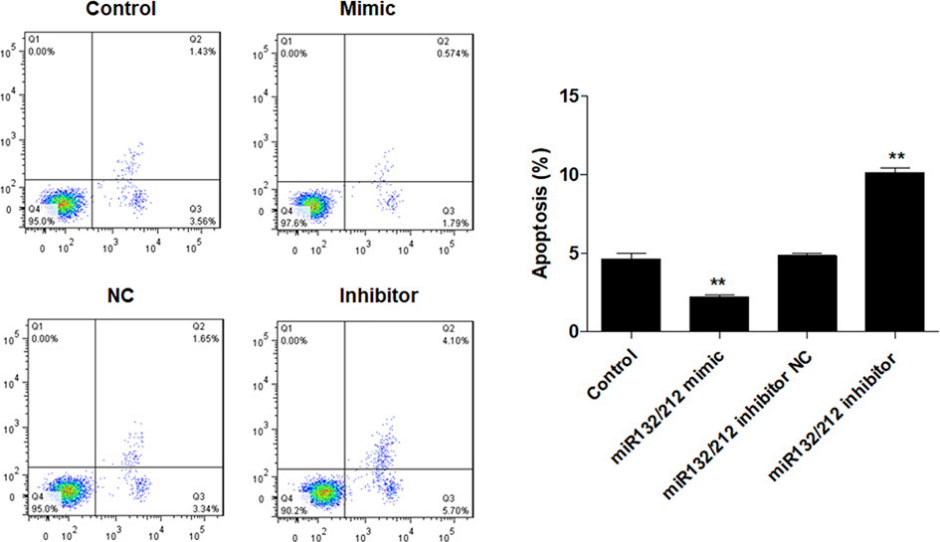
miR-132/212 cluster suppresses apoptotic death in PCSCs After transfection with miR-132/212 mimic, inhibitor and negative control (NC), flow cytometric analysis was performed to measure the cell apoptosis of PCSCs; *P<0.05, **P<0.01.

It is well established that cell apoptosis is closely associated with proliferation ability. Thus, we further examined the effect of miR-132/212 cluster on PCSCs apoptosis. Cytometry analysis showed that overexpression of miR-132/212 cluster significantly suppressed the numbers of apoptosis in PCSCs compared with negative controls, while down-regulation of miR-132/212 cluster elevated the apoptotic cell number in PCSCs (Figure 3). Moreover, miR-132/212 inhibitor NC had no obvious effects on PCSCs apoptosis. Taken together, these data showed that miR-132/212 cluster promotes PCSCs growth through inhibition of apoptosis.

### PTCH1 is a direct target of miR-132/212 cluster

**Figure 4:**
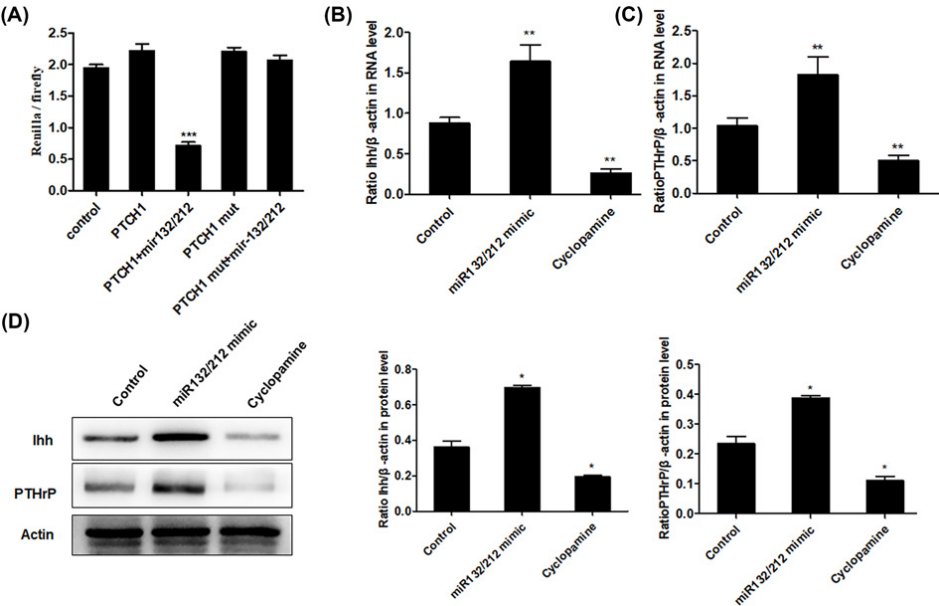
miR-132/212 targets PTCH1 and regulates Ihh/PTHrP signaling pathway Luciferase reporter assay was used to validate that PTCH1 was a direct target of miR-132/212 (**A**). The mRNA and protein levels of Ihh and PTHrP were detected by real-time PCR (**B** and **C**) and Western blot (**D**), and then quantified to the control β-actin; *P<0.05, **P<0.01.

Bioinformatics analysis using online tools, including miRanda, PicTar and TargetScan, was performed to identify potential targets of miR-132/212 cluster. As a result, the 3′UTR of PTCH1 gene was found to contain the conserved binding sites for miR-132/212 cluster. To further verify that PTCH1 is a potential target of miR-132/212 in PCSCs, we generated luciferase reporters that contained the 3′UTR or a mutated sequence within the biding site of PTCH1 gene. Consequently, dual luciferase reporter assay showed that the activity of wild-type PTCH1-3′UTR was significantly decreased in the presence of miR-132/212 cluster. However, the luciferase activity of mutated PTCH1-3′UTR remained unchanged after co-transfection with miR-132/212 cluster (Figure 4A). In addition, real-time PCR (Figure 4B,C) and Western blot (Figure 4D) showed that the mRNA and protein expression of Ihh and PTHrP was significantly elevated following treatment with miR-132/212 mimics and decreased after treatment with cyclopamine, an inhibitor of Hh pathway. Taken together, these findings validated that PTCH1 was a direct target gene of miR-132/212 in PCSCs, and mediated the activation of Ihh/PTHrP signaling pathway.

### Cyclopamine inhibits growth and promotes apoptosis of PCSCs

**Figure 5:**
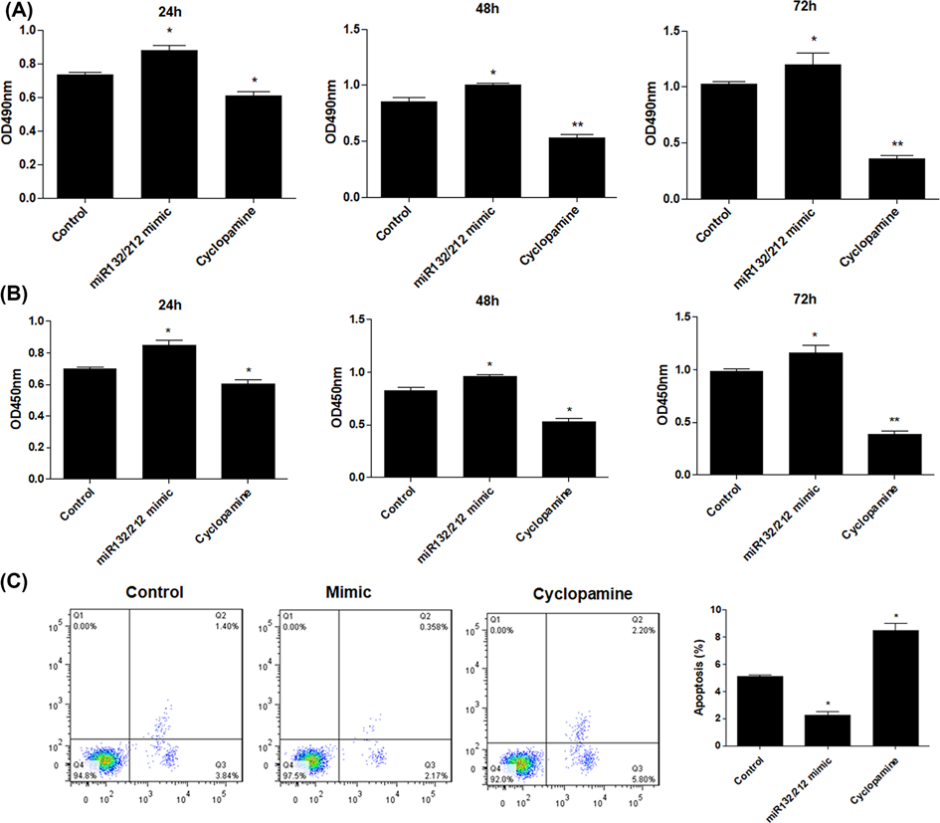
Cyclopamine inhibits growth and promotes apoptosis of PCSCs After treatment with cyclopamine, MTT assay (**A**) and BrdU assay (**B**) were performed to measure the cell viability and DNA synthesis of PCSCs at 24, 48 and 72 h. Moreover, flow cytometry (**C**) was performed to measure the cell apoptosis of PCSCs; *P<0.05, **P<0.01.

Furthermore, we investigated the role of cyclopamine in the cell viability of PCSCs. MTT analysis showed that miR-132/212 mimic transfection increased cell viability of PCSCs at different time points. By contrast, cyclopamine treatment suppressed PCSCs growth (Figure 5A). In addition, BrdU assay confirmed that up-regulation of miR-132/212 cluster promoted the DNA synthesis, which was reversed treatment with cyclopamine (Figure 5B). Cytometry analysis showed that overexpression of miR-132/212 cluster significantly suppressed the numbers of apoptosis in PCSCs. Conversely, cyclopamine treatment elevated the apoptotic cell number in PCSCs (Figure 5C).

## Discussion

PCSCs could self-renew or differentiate into chondrocytes to promote bone growth [2]. Through immunomagnetic beads, PCSCs were successfully isolated, purified and cultured from the perichondrial mesenchyme of New Zealand white rabbits. In the present study, we for the first time demonstrated that miR-132/212 cluster contributes to the proliferation and inhibits apoptosis of PCSCs by targeting Ihh/PTHrP signaling pathway. Our evidence suggests that Ihh/PTHrP signaling cascade is important for miR-132/212-induced growth in PCSCs.

Studies have shown that miRNAs play an important role in regulating multiple developmental processes in diverse organisms such as stem cell chondrogenic differentiation for osteogenic differentiation or articular cartilage regeneration [13,14]. The biological function of miR-132 and miR-212 may be different although they share the same seed sequence [15]. Studies have shown that miR-132 promotes proliferation and migration of human umbilical venous endothelial cells and vascularization in animal models [12]. Some research indicated that miR-212 is elevated in lung cancer, leading to abnormal cell cycle progression, apoptosis and migration [16]. A recent study has reported that miR-132 and miR-212 cluster function as a tumor suppressor in thyroid cancer cells by CSDE1 mediated post-transcriptional program [17]. Other studies in non-tumor disease report that overexpression of miR-132 and miR-212 promote endothelial cell growth, leading to an enhanced vascularization under ischemic condition [12]. It also has been observed that miR-132 could promote chondrogenic differentiation of rat mesenchymal stem cells [18]. However, the effect of the miR-132/212 cluster on the growth of PCSCs remains unclear. In the present study, we found that enforced expression of miR-132/212 significantly increased the proliferation and DNA synthesis, and inhibited apoptosis in PCSCs. In contrast, inhibition of miR-132/212 decreased cell growth and DNA synthesis, and promoted apoptosis in PCSCs. Taken together, our study showed that miR-132/212 cluster is beneficial for the growth of PCSCs, representing its potential role in cartilage regeneration.

The Hedgehog (Hh) signaling pathway is evolutionarily conserved and plays important roles in homeostasis and development. Deregulation of Hh signaling results in a wide range of human diseases [19]. Particularly, accumulating evidence indicates the critical role of Hh signaling in bone development, homeostasis and repair, such as limb patterning, endochondral ossification and joint formation [20,21]. Abnormalities of the Hh pathway lead to various bone diseases. A previous study showed that mutations in Ihh leading to human brachydactyly type A1 characterized by shortened middle phalanges [22].

PTCH1 is a transmembrane protein that forms a receptor complex for Shh together with Smoothened. The Ptch1 protein is expressed in the cell membrane of target tissues, where it represses Smoothened pathway [23]. It has been shown that mutational inactivation of PTCH1 results in the activation of the Hh pathway [24]. Moreover, PTCH1 mutations can cause nevoid basal cell carcinoma syndrome, in which bone abnormalities include rib anomalies, polydactyly, spina bifida and ectopic ossification [25]. In the present study, the putative binding site of miR-132/212 in the 3′–UTR region of PTCH1 was identified by bioinformatic analysis. Luciferase reporter assays indicated that transfection of miR-132/212 led to a marked reduction of luciferase activity, but had no effect on PTCH1 3′-UTR mutated fragment. In addition, Western blot showed that the protein expression of Ihh and PTHrP, components of Hh pathway, was significantly elevated following treatment with miR-132/212 mimics and decreased after treatment with cyclopamine, an inhibitor of Hh pathway [26]. Furthermore, inhibition of Ihh/PTHrP signaling by cyclopamine significantly suppressed growth and DNA synthesis, and induced apoptosis in PCSCs. By contrast, miR-132/212 mimic transfection increased PCSCs growth and inhibits cell apoptosis. Taken together, these results suggested that miR-132/212–induced promotion of PCSCs growth was mediated by targeting PTCH1 and activating Ihh/PTHrP signaling pathway.

In summary, the present study provides new insights into the role of miR-132/212 in PCSCs growth. Our findings demonstrate that miR-132/212 potently promotes PCSCs growth and inhibits apoptosis by regulating PTCH1-mediated Ihh/PTHrP signaling pathway, suggesting that miR-132/212 cluster might serve as a novel target for tissue regeneration and repair.

## Abbreviations

DMSO: dimethyl sulfoxide
FGFR-3: fibroblast growth factor receptor-3
Hh: Hedgehog
Ihh: Indian hedgehog
miRNA: microRNA
NC: Negative control
PCSC: precartilaginous stem cell
PTCH1: Patched 1
PTHrP: Parathyroid hormone related protein
UTR: untranslated region
